# The missing middle service gap: Obtaining a consensus definition of the ‘Missing Middle’ in youth mental health

**DOI:** 10.1177/00048674241299221

**Published:** 2024-11-27

**Authors:** Jana M Menssink, Caroline X Gao, Isabel Zbukvic, Sophie Prober, Athina Kakkos, Alice Watson, Sue M Cotton, Kate M Filia

**Affiliations:** 1 Faculty of Medicine, Dentistry and Health Sciences, University of Melbourne, Parkville, VIC, Australia; 2 Orygen, Parkville, Melbourne, VIC, Australia; 3 Department of Epidemiology and Preventive Medicine, Monash University, Clayton, VIC, Australia

**Keywords:** Youth mental health, delphi study, missing middle, mental health, care gaps, missing middle service gap, treatment gap, service gap

## Abstract

**Objective:**

As highlighted in Australia’s Productivity Commission Inquiry into mental health, subgroups of individuals are failing to have their needs met, or are ‘falling through the cracks’ in the current system – a phenomenon increasingly referred to as the ‘missing middle’. A barrier to devising solutions is that the term ‘missing middle’ is not clearly defined. Using the Delphi method, we aimed to define the term and explore acceptability.

**Method:**

Three expert groups were recruited: carers and young people with a lived experience of mental ill-health; clinicians and service providers; researchers, policymakers and commissioners of mental health services. Using a three-stage Delphi process, we elicited definitions, refined and developed a consensus definition.

**Results:**

Ten subthemes describing the ‘missing middle’ were identified, with four endorsed across all expert groups from the outset: service gap, inflexibility, inadequate service quality and duration, and social disadvantage. Additional subthemes were later endorsed. Feedback was sought on a consensus-driven definition that encompassed the original four endorsed subthemes. Findings supported a shift to a systemic focus – framing the ‘missing middle’ as a care gap.

**Conclusions:**

A consensus definition was developed, repositioning the term to a systems lens, describing a ‘missing middle service gap’. The definition represents the ‘missing middle’ as a term to describe a gap in care where existing mental health services are not meeting the needs of individuals in a meaningful way. Research was carried out in relation to youth mental health in Australia and the definition may need to be adapted for other contexts.

The ‘missing middle’ is a term with a variety of definitions in multiple contexts, spanning economics, global aid and development, and healthcare sectors (Hsieh and Olken, 2014; Veldhuizen et al., 2020). The term ‘missing middle’ in the mental health context is mainly used in Australia and the United Kingdom. In Australia, it was adopted during the 2019 Productivity Commission Inquiry into Mental Health (Productivity Commission, 2019) and the 2021 Royal Commission into Victoria’s Mental Health System (RCVMHS, 2021) in reference to unmet care needs in the mental health system. It has also been an important part of discourse on the mental health impacts of the COVID-19 pandemic, given the increased prevalence of mental ill-health and exacerbation of pre-existing issues, with individuals experiencing prolonged stressors and social isolation (McGorry, 2022; Samji et al., 2022). While utilisation of the term varies, it is most commonly used to refer to those who require more specialised care than available within primary, low-to-moderate intensity care providers (‘too unwell’), but do not meet eligibility for specialist and higher-intensity care (‘not unwell enough’; Neagle et al., 2018; Fava, 2021; O’Reilly et al., 2022; Petrie et al., 2021; Productivity Commission, 2020; Savaglio et al., 2023, 2024; Thomas et al., 2022). The ‘missing middle’ has also been used to describe individuals who are moderately unwell but still partially functioning, who do not meet eligibility criteria for publicly funded treatment and do not have the financial means to access required care privately (Burchett and Elford, 2022; Neagle et al., 2018; Petrie et al., 2021). The study by Petrie et al. (2021) with 570 health practitioners led them to describe the ‘missing middle’ as individuals who were both: (1) not ‘severe enough’ for assistance or (2) too severe for ‘program A’ but not severe enough for ‘program B’ (i.e. not eligible for any assistance). The ‘missing middle’ has also been used to articulate the consequences of this gap, such as a worsening of conditions, sometimes to the point of requiring emergency or hospital treatment (McGorry and Mei, 2020; Petrie et al., 2021; Thomas et al., 2022). There is a clear service system context, but lack of consensus is a barrier for advocates attempting to advise policy makers and service providers on the need to respond to this service gap.

In Australia, ‘missing middle’ advocacy has been spearheaded by youth mental health expert Prof. McGorry (2022), in light of increasing service needs and corresponding lack of resources for young people. Advocacy has helped raise awareness; however, the term ‘missing middle’ is continually used in varying ways, with some commonalities but also clear differences (Duckett and Swerissen, 2020; Dunn et al., 2024; Nette, 2019; Fava, 2021; Petrie et al., 2021; Savaglio et al., 2023). For example, while some refer to the ‘missing middle’ as a cohort characterised by not meeting service eligibility, descriptions may also include those who are underserved (i.e. engaged in suboptimal care when needing specialised services; Fava, 2021), whereas others refer to service gaps: ‘the missing middle of service provision’ (Nette, 2019; Productivity Commission, 2020). Moreover, definitions are often supplemented with various suggested psychosocial characteristics (e.g. illness chronicity, comorbidities, social exclusion) and/or service needs (e.g. requiring long-term, multidisciplinary care; Dunn et al., 2024; McGorry, 2022; Savaglio et al., 2023). Inconsistencies make it difficult to articulate issues with clarity and hamper empirical research on the ‘missing middle’ concept, which in turn impedes the design of effective research and solutions (Looi et al., 2022). Clearly, addressing these research gaps and building a stronger foundation of knowledge is necessary to develop evidence-based policy, mental healthcare planning and service implementation to address unmet needs.

The aim of the study was to develop a definition of the term ‘missing middle’ in the context of youth mental health in Australia by obtaining a consensus from three groups: (1) youth and carers with a lived experience; (2) clinicians and service providers; and (3) researchers, policy makers and commissioners of mental health services. Building on this, further aims included: (1) exploring acceptability of the term as it is currently used and (2) identifying whether the consensus term developed was acceptable to our expert groups.

## Method

### Design

The Delphi technique is an ideal methodology for developing an expert consensus on a novel term (‘missing middle’) from a broad range of stakeholders (Avella, 2016). Delphi studies work optimally with heterogeneous groups (Jorm, 2015), lending themselves to capturing diverse expertise; this is vital in mental health research, where consumers, carers, clinicians, researchers and policy makers are all ‘experts’ and have valuable and differing perspectives. Conducting consensus work online allows for diverse geographical representation, which is important when considering issues of service availability and accessibility.

Ethical approval for the study was obtained from The University of Melbourne Human Research and Ethics Committee (HREC) in 2020 (2056430).

### Sample and setting

Participants were recruited through social media and targeted emails (e.g. contacting commissioners at Primary Health Networks [PHNs]). We recruited three groups of participants: (1) youth (aged 16–25) with lived experience of mental illness and caregivers (lived experience expert group); (2) clinicians and service providers (clinical expert group); and (3) commissioners of mental health services within PHNs, researchers and policy makers with expertise in the field of youth mental health (health service [HS] expert group). Participants were deemed eligible for the lived experience expert group if they had a lived experience of mental illness and were aged 16–25 years old or were a carer of this group (Law and Morrison, 2014). Clinical and HS expert groups were eligible if they had at least 1 year of experience working in the field of youth mental health (Richards et al., 2022).

### Measures

#### Demographic variables

Demographic information was collected including age, gender, residential postcode and country of birth. Participants were asked whether they had a lived experience of mental ill-health, their primary expert role, and if applicable, how many years they had spent working in mental health.

#### Delphi questionnaire

In Stage 1, we posed four open-ended questions to participants: (1) ‘What do you think the *missing middle* means in the context of youth mental health?’; (2) ‘What characterises someone as a part of the *missing middle*?’; (3) ‘Do you feel the term conceptualises the population accurately? Is there another way you would refer to this group?’; (4) ‘Are there different subgroups within the *missing middle*?’ Given those with lived experience were ‘experts by experience’ (British Psychological Society, 2000) and may be unfamiliar with the terminology ‘missing middle’, we oriented participants to a broad, layperson definition prior to the survey:

‘*individuals who are not having their needs met or are “falling through the cracks” in the mental health system . . . Based on the current general understanding of the “missing middle”, there is a substantial proportion of young people with mental illness who are not receiving adequate treatment. It is thought that a combination of factors has contributed to this*’.

Following a thematic analysis of responses (*n* = 147), the Delphi questionnaire was developed, consisting of 12 items (statements) categorised into three domains, for consensus rating in Stages 2 and 3 (detailed below).

#### Endorsement criteria

In a Delphi study, items are rated in each round and meet criteria for ‘endorsement’, ‘re-rating’ or ‘neither’. Stages are repeated until a substantial consensus (e.g. greater than 80% for each group) is reached. To inform re-rating, participants are provided a feedback report, consisting of anonymous group ratings for each statement (e.g. 75% agree, 25% disagree) and given the option to change their previous response. We selected commonly used cut-off criteria: items achieving positive agreement ratings (Moderately or Very important) of greater than 70% in two or more groups, or of at least 80% of one group, are included in the following round for re-rating. Items with lower agreement ratings are discarded.

### Procedure

In Stage 1, participants were invited via email or social media to review the Plain Language Statement and Consent form; consenting participants completed a survey via Qualtrics consisting of open-ended questions to explore the acceptability of the term as currently used, and to compile characteristics and subjective definitions of the ‘missing middle’.

In Stage 2, the project was advertised again to bolster sample size and minimise the effects of dropout at each round. The expert panels were asked to read the items provided, add any statements they felt were missing, and rate the importance of each item in defining the ‘missing middle’ on a 4-point Likert-type scale: Not important, A little important, Moderately important, Very important. Responses were analysed to determine the rate of agreement, and themes were either endorsed, discarded or required re-rating as per endorsement criteria outlined above.

In Stage 3, items to be re-rated were presented to participants. An excerpt from the feedback report is displayed in Supplementary Figure 1. Participants were asked to provide comments and rate the validity of a draft definition using a Likert-type scale of five items: Inaccurate, Somewhat inaccurate, Neutral, Somewhat accurate, Accurate.

### Data analysis

The first stage of data analysis involved reflexive thematic analysis with coding in line with the guidelines by Braun and Clarke (2021). The first author and coder is a researcher and psychologist with 7 years of experience working clinically with adults, young adults and adolescents. The second coder is a research assistant in youth mental health and is within the age range of our youth participant group.

Quantitative analysis was performed using R (v 4.1.0) for simple descriptive statistics to examine the demographics and total percentage agreements.

## Combined results and discussion

### Sample characteristics

Participant characteristics across all three stages are displayed in Table 1. One hundred and forty-seven people participated in Stage 1. Forty-nine records were excluded due to incomplete data and substantial missingness (> 50% of data missing) for a range of variables. Most participants were from Victoria (37%) and New South Wales (24%), thereafter Queensland (13%) and Tasmania (12%) and only a few were from South Australia (5%), Northern Territory (5%) and Western Australia (4%). Responses (*n* = 98) were analysed and synthesised based on salience into three themes and ten subthemes, rated in Stage 2.

**Table 1.** Demographic characteristics of participants in each of the three stages of the Delphi study.

		Stage 1 (*n* *=* 98)	Stage 2 (*n* *=* 168)	Stage 3 (*n* *=* 105)
Demographics				
Age	Mean age (*SD*)	36.8 (13.0)	38.4 (13.0)^a^	37.8 (14.0)
	Age range (min–max*)*	17–72	16–72	16–72
Gender	Female % (*n*)	74.5 (73)	76.2 (128)	77.1 (81)
	Male % (*n*)	23.5 (23)	20.2 (34)	19.0 (20)
	Non-binary or other % (*n*)	2.0 (2)	3.6 (6)	3.8 (4)
Born in Australia % (*n*)		70.4 (69)	66.7 (112)	75.2 (79)
Aboriginal or Torres Strait Islander % (*n*)		3.1 (3)	1.2 (2)	1.9 (2)
Lived experience self-report				
	Experience of mental illness (current or past) % (*n*)	59.2 (58)	48.8 (82)	61.9 (65)
	Experience as carer (current or past) % (*n*)	27.5 (27)	26 (43)	29.5 (31)
	Prefer not to answer % (*n*)	5.1 (5)	3.0 (5)	3.8 (4)

a Three participants did not provide age.

In the lived experience expert group, there was relatively high retention across all stages; however, between Stages 2 and 3, there was a 50% dropout in the clinical expert group and ~39% in the HS expert group (see Table 2).

**Table 2.** Participation rates in the three stages of the Delphi study.

Stages	Expert groups		
	Lived experience % (*n*)	Clinical % (*n*)	HS % (*n*)
Stage 1 *n* = 98	34.7 (34)	48.9 (48)	16.3 (16)
Stage 2 *n* = 168	27.4 (46)	46.4 (78)	26.2 (44)
Stage 3 *n* = 105	37.1 (39)	37.1 (39)	25.7 (27)

HS: health service.

### Stage 1 findings

Fifty-seven percent of participants (*n* = 56) had heard of the term ‘missing middle’. Nineteen percent (*n* = 19) identified as being part of the ‘missing middle’ themselves. In Stage 1, approximately half (49.4%) of all participants identified as experiencing mental illness currently or in the past, and a quarter (25.9%) were, or had been, a carer for someone with mental ill-health. Many who selected their primary role as HS or clinical expert also had experience of mental ill-health and/or as a carer. Years of experience working in mental health ranged from 1 to 40 (*M* = 11.1, *SD* = 9.3).

*Baseline acceptability of the term missing middle.* At the outset, study participants were asked about the acceptability and appropriateness of how the ‘missing middle’ term is currently used in their spheres. There were three key themes identified in participant responses: acceptable, unacceptable and mixed. The mixed theme refers to deeming the term acceptable overall with some caveats or concerns.

Acceptable: Forty percent of participants expressed that the ‘missing middle’ accurately captured the concept, viewing it as useful and innocuous: ‘*I think it is a good term . . . I think it refers to missing out on services, or un-serviced middle, rather than a pejorative term*’ (ID46, Clinician). They described it as acceptable and reflecting their personal experiences accurately: ‘*Having lived and sought treatment for an adolescent who was aware he had problems, I think the term encapsulates the situation very well*’ (ID70, Carer).

Unacceptable: Approximately a third (32%) said the term ‘missing middle’ was inaccurate, disrespectful, overly simple or lacking in clarity: ‘*No, it is too vague. “* ***Mental illness sufferers without adequate care****” describes it better but doesn’t have the cutesy ring to it, and it highlights Australia’s funding and services are not good enough*’ (ID74, Young person, emphasis added).

Some found the term upsetting and offensive when used to refer to individuals or groups. Suggestions were made that there should be a shift to focus on systems and services:

*This term should refer to services and service design, not to people. I find it distressing when I hear people say that (the person I care for) is part of the missing middle. It is the system that has this problem, not him* (ID16, Carer).

Others did not necessarily see the ‘missing middle’ as a negative term, instead viewing it as unacceptable because it was inaccurate or lacked utility when referring to people: ‘*I think it’s a terrible term and used as a catch all*’ (ID43, PHN).

Mixed: About a third (28%) said the term was acceptable but believed there was room for improvement and/or flagged concerns with it being used inappropriately at times: ‘*The term is probably wrapped up in current funding arrangements (the Medicare-state dichotomy) rather than reflecting the actual mental health presentations or needs of these young people . . .* ’ (ID99, Researcher). Participants suggested that when the term is used to refer to individuals, it does not capture the fluctuating nature of care needs or changes in stage of illness over time.

*The term is accurate in some ways . . . but is perhaps too static. It needs to be remembered that those considered missing middle are on shifting trajectories and are not those who have never had services; they may have become too complex for one service but be denied another (failure to step-up) or are prevented from stepping down as they are no longer early intervention or have residual symptoms and function decline* (ID34, Clinician).

Some participants reported that while they had concerns about its appropriateness, it worked well in facilitating communication: ‘*. . . However, I understand the missing middle allows for a complex idea to be easily communicated*’ (ID24, Young person).

Within the theme Mixed, a notable subtheme was of the position that other labels are needed to capture the different groups with unmet care needs: ‘*. . . those who are deemed as not being deserving of funding for any form of support (people on particular visas) could be another category entirely. Maybe we could call them the invisibles?*’ (ID46, Clinician).

Across expert groups it was asserted that the term is more useful to describe service gaps and it is erroneous to apply this label to a diverse and dynamic group of individuals: ‘*It's such a big heterogeneous group that there's no one term which would cover everyone. It's a useful way to refer to a service gap, but not a useful way to refer to a client group*’ (ID57, Researcher). Others suggested the term was misused at times, for example, positing that the ‘missing middle’ should not be used to refer to groups with unmet care needs due to poor health literacy.

### Impacts: secondary stress

Participants also spoke about how they were impacted by the ‘missing middle’. Disclosures were unprompted and suggest that individuals wanted to share challenges experienced due to the ‘missing middle’. Impacts, largely related to secondary stress, were reported by carers, young people and clinicians: ‘*I still see a psychologist to manage what we've gone through with our son*’ (ID133, Carer). Stress related to difficulty affording ongoing healthcare costs was also described: ‘*Assumptions we can afford to pay for ongoing services (it's been nearly 3* *years now) . . . [and] about our capacity as a family to protect and support our child, regardless of how unwell he was/is*’ (ID133, Carer). Some reported feeling demoralised by the lack of available services and concern about the impacts of a downstream approach of care, expressing significant worry about the health deterioration of those whose care needs are unmet:

*Personally at times I have felt very frustrated, sad and hopeless as I can see these young people who are not able to receive the care and support they need and deserve. I then fear that they will keep presenting to emergency departments, continue to get marginalised, face further disadvantages, and their mental health will continue to decline until they are finally deemed ‘suitable’ for support . . . where early intervention, or appropriate supports could have prevented this* (ID118, Clinician).

### Themes and preliminary definition based on Stage 1 findings

Initial coding revealed three major themes: systems, psychosocial and barriers. Each was distinct in how the ‘missing middle’ was conceptualised: (1) a systemic issue within existing mental health services; (2) a term to describe a group of people with complex psychosocial circumstances including severe and/or comorbid illnesses, social exclusion and requiring multidisciplinary types of care, longer or more intensive care; and (3) barriers to treatment access. Within these three major themes, 21 subthemes were identified. Those that had considerable overlap were combined, then narrowed down to 10 subthemes (see Table 3) based on saliency (i.e. themes were removed if endorsement was *n* < 20).

**Table 3.** The 10 subthemes identified in Stage 1 of the Delphi study.

The ‘missing middle’ is . . . a term to describe a systemic issue where there is a(n):	
1	Service gap^a^: The lack of available services to assist young people who do not qualify for primary care (e.g. treating mild to moderate illness) or tertiary care (e.g. treatment for severe, acute illness that may involve hospitalisation)
2	Inflexibility in services: Inflexible services bound by rigid criteria; unable to adapt to accommodate a young person’s care needs and circumstances. For example, imposing overly rigid eligibility criteria for access to and continuity of care (e.g. discontinuity of care for those who move to different catchment areas, with those in unstable housing potentially disproportionately affected)
3	Inadequate service quality and duration: The appropriate type, intensity and duration of treatment are not provided in a timely way (e.g. limit of Medicare subsidised sessions, or type of evidence-based treatment required is not widely available)
The ‘missing middle’ is . . . a term to describe a group of young people who experience:	
4	Chronic mental illness: Longstanding and persistent mental health challenges requiring long-term care
5	Comorbidities: Two or more co-occurring mental health, physical health, substance use and/or behavioural issues
6	Social disadvantage: A young person from a group or community that is at higher risk of poor health due to vulnerabilities resulting from a range of economic, social, physical and psychological factors, such as homelessness, belonging to CALD, LGBTIQ+ and/or forensic populations
7	Adverse experiences: Stressful life events or trauma that can have an enduring impact on mental health (e.g. abuse). This includes but is not limited to stressful events that occurred during childhood (e.g. having a parent with mental illness)
The ‘missing middle’ is . . . a term to describe barriers to treatment access due to:	
8	Lack of resources: Reduced access to mental health services due to limited personal resources. For example, not having the finances, time or transport required to access care (i.e. experiencing socio-economic disadvantages, living in rural or remote regions)
9	Under-recognition: Their mental ill-health going unrecognised, for example, because symptoms are attributed to another cause, their condition is not seen as ‘severe enough’, and/or are at risk but do not meet diagnostic criteria for mental illness
10	Engagement issues: Where participation in healthcare services is difficult or decreased due to prior negative experiences with the system (e.g. long waitlists, continuously referred on from service to service), or support is not flexible enough to accommodate for barriers or others issues that may interfere with access to treatment

CALD: culturally and linguistically diverse; LGBTIQ+: lesbian, gay, bisexual, transgender, intersex, queer or questioning and more.

a Service gap conceptualisation is now positioned to focus on the system rather than the individual.

Interrelationships were evident between several of the subthemes, for example, inflexibility in services and engagement issues had significant overlap, with the following quote illustrating how inflexibility in services contributes to disengagement: ‘*Those young people who do not quite fit in criteria for different services, are referred on and on until they give up seeking help*’ (ID72, Clinician).

Under-recognition had some overlap with Inflexibility of services: ‘*People who aren’t getting adequate help because they don’t fit the right “boxes.” E.g. don’t meet enough criteria to fit the diagnosis of a serious mental health disorder . . . but still having serious mental health issues*’ (ID14, Young person). Under-recognition also captured mis- and under-diagnosis, with the quote below illustrating increased risk of under-recognition for marginalised communities and overlap with comorbidities:

*I feel the discourse about the missing middle fails to bring awareness about mis/underdiagnosis of neurodivergent conditions like ASD and ADHD, particularly for women and CALD communities . . . I wasn't able to keep my mood disorders and anxiety under control until I was properly treated for ADHD. I wouldn't have [been] stuck in the missing middle for 6 years if I was properly diagnosed . . . my perceived giftedness and my cultural background made it easy for adults to overlook and ignore my difficulties*. (ID159, Carer and Young person).

## Stage 2 findings

In Stage 2, participants rated the importance of the 10 subthemes for the definition of the ‘missing middle’. Participants endorsed four subthemes as most important in defining the ‘missing middle’: service gaps, inflexibility, inadequate service quality and duration, and social disadvantage.

There were some differences between expert groups for the remaining six subthemes (see Table 4). For example, those with a lived experience rated under-recognition more highly (89%) than other groups (72% and 73%). Based on the four endorsed subthemes, a draft definition was developed by the authors:

*The missing middle refers to systemic issues in the mental health care system, which lead to unmet care needs of young people experiencing mental health problems. Systemic issues can include the gap between primary and tertiary services, inflexibility of services (e.g. rigid eligibility criteria) affecting access to and continuity of care, and inadequate service availability in terms of the quality of service (i.e. lack of specialised services) or duration of treatment. These systemic issues disproportionately affect youth with social disadvantage or marginalised identities (CALD, unstable housing)*.

**Table 4.** Expert group ratings of the importance^a^ of the 10 subthemes in Stage 2.

Expert group Domain	Clinical (*n* = 46) %	Lived experience (*n* = 78) %	Knowledge (*n* = 44) %
Service gap	98.7	88.9	97.7
Inflexibility	91.0	95.5	86.4
Inadequate service quality	93.6	93.3	90.9
Chronic mental illness	73.1^b^	82.6	56.8^b^
Comorbidities	80.8	71.7^b^	77.3^b^
Social disadvantage	80.8	82.6	86.4
Adverse experiences	87.2	80.4	75.0^b^
Lack of resources	88.5	76.1^b^	72.7^b^
Under-recognition	71.8^b^	89.1	72.7^b^
Engagement issues	85.9	89.1	77.3^b^

a Moderately important and very important combined.

b Below 80.

## Stage 3 findings

In Stage 3, participants re-rated six subthemes and provided feedback on the accuracy of the draft definition. After some participants changed their ratings, all six subthemes were endorsed (see Table 5).

**Table 5.** Expert group ratings of the importance of subthemes re-rated in Stage 3.

Domain	Clinical (*n* = 39)	Lived experience (*n* = 39)	HS (*n* = 27)
	%	%	%
Chronic mental illness	79.5	89.7	70.4^a^
Comorbidities	84.6	89.7	85.2
Adverse experiences	89.7	87.2	88.9
Lack of resources	89.7	94.9	81.5
Under-recognition	84.6	100.0	77.8^a^
Engagement issues	84.6	94.9	88.9

HS: health service.

a Below 80.

While these six subthemes were rated as important for inclusion, the draft definition consisting of only four subthemes endorsed in Stage 2 had substantial support. Overall, 97.1% of participants rated it as somewhat accurate (35.6%) or accurate (61.5%), as outlined in Table 6. No one rated the definition as inaccurate. In addition, some participants provided more detailed feedback in response to open-ended questions, including several positive remarks (*n* = 6) about the systems lens: ‘*This is a good definition so far and I like that it [is] contextualised as a systemic issue rather than a focus on the person or diagnosis as the problem*’ (ID131, PHN).

**Table 6.** Expert groups’ perceptions of accuracy of the draft definition of the missing middle in Stage 3.

Accuracy	Clinical (*n* = 39)	Lived experience (*n* = 39)	HS (*n* = 27)	Total (*N* = 105)	*p*-value
	*n* (%)	*n* (%)	*n* (%)	*n* (%)	0.420
Accurate	26 (68.4)	24 (61.5)	14 (51.9)	64 (61.5)	
Somewhat accurate	11 (28.9)	13 (33.3)	13 (48.1)	37 (35.6)	
Somewhat inaccurate	1 (2.6)	2 (5.1)	0 (0.0)	3 (2.9)	
Missing data (*n*)	2	0	4	6	

HS: health service.

Eight participants suggested that either a more succinct definition or change in language was needed: ‘*I have concerns about the term “marginalised identities.” Maybe consider “who may be marginalised by community” or “face discrimination”*’ (ID138, Clinician).

The authors revised the draft definition to include the additional characteristics endorsed during Stage 3 and to incorporate feedback from participants. Wording was revised for succinctness and the two subthemes ‘Social disadvantage’ and ‘Lack of resources’ were included under the umbrella term ‘social exclusion’ (Filia et al., 2018). Inflexibility of services and Engagement issues were similarly combined given the overlap in meaning whereby rigid procedures and requirements to qualify for services were described as interfering with access to services. The definition was further refined to enhance clarity and generalisability by avoiding use of context-specific terms such as primary and tertiary healthcare. These steps led to the following definition being developed:

The ‘missing middle’ describes systemic gaps in care where mental health services do not meet the needs of individuals in a meaningful way. Structural issues include a gap between services designed to treat mild mental illness and services designed for acute or severe mental illness, inflexibility of services (e.g. rigid eligibility criteria affecting engagement and continuity of care), and inadequate treatment (limited availability, suitability and/or duration). Missing middle service gaps may disproportionately impact individuals affected by social exclusion, traumatic and adverse life experiences, chronic and/or comorbid illnesses.

## General discussion

This study is significant in drawing on the expert viewpoints of multiple stakeholder groups with a mutual intention to reach a shared definition of the missing middle. A systems-based definition of the missing middle was most highly endorsed across all groups from the outset, and in Stage 3 when rating the sample definition, which mainly represented systemic focused themes, most participants (97.1%) provided high accuracy ratings: ‘*It's perfect. It helps to name the SYSTEM as the missing middle, rather than the term being like a diagnosis of a person or group*’ (ID211, PHN). The reframing to a ‘missing middle service gap’ may seem subtle in some respects, but it shifts the onus from the individual to the system. It also reduces the risk of inadvertently placing expectations on individuals and carers to navigate and try to access difficult-to-reach or unavailable care.

Our findings align with the definition used by the Productivity Commission (2020), which posits that ‘the *missing middle gap* is a service gap’, where individuals do not meet eligibility for primary or specialised mental health services, nor are able to access alternative services due to long waitlists or unaffordable out-of-pocket costs, with added barriers faced by groups experiencing discrimination and those living outside major cities (p. 46, Vol 1; Productivity Commission, 2020). Our results extend on this definition, suggesting there are additional barriers to access in the youth mental health system context, such as inflexible services, which do not adapt to meet young people’s care needs and circumstances. This is perhaps unsurprising, given the unique care needs of young people and the risk of falling through service gaps between child and adolescent services and adult services faced by young adults (aged 18–25 years; McGorry et al., 2014). As one young person stated: ‘*Being told you are too complex for services like [youth mental healthcare provider] but not being helped by adult public services*’ (ID14, Young person). In combination with the findings from the Productivity Commission (2020), the present findings demonstrate the relevance of the missing middle service gap across youth and adult mental health service systems, adding to the evidence and the case for policy action to address this issue.

The development of a consensus definition has been an important first step. One of the next challenges in addressing missing middle service gaps relates to identifying and learning from those who are missing out (service exclusion) or who are engaged in services that are not well-matched to their care needs (under-served). As illustrated by the findings, service exclusion may be intentional (i.e. rigid eligibility criteria) or unintentional (i.e. costs are unaffordable; Ng et al., 2022). The subtheme ‘Inadequate service quality and duration’ showed the multiplicity of the issue of being under-served:

*Young people with mental illness whose service needs are not being met in terms of the frequency (number of sessions); breadth (access to a range of providers working together to take a holistic approach to care); and quality of care (evidence-based interventions, well-trained and supported clinical and non-clinical service providers)* (ID115, HS expert).

Ascertaining how best to identify those affected by missing middle service gaps is necessary for both making and evaluating progress.

### Strengths and future directions

The Delphi methodology provided a platform for people, including youth and caregivers, to provide their perspectives without having to navigate perceived power differentials or conflicting opinions. It is important that those affected by missing middle service gaps are involved in sharing their expert knowledge, with their voices contributing directly to our understanding of the missing middle, and guiding future research directions and integrated service system responses (ACT Health Directorate, 2022; Kaine and Lawn, 2021). Moreover, the conceptualisation of the missing middle as a service gap avoids the social construction of a group of ‘dependents’ who are typically framed as powerless or helpless (Schneider and Ingram, 1993).

Our findings showed a shift away from a definition, which some participants found inaccurate and overly simplified: ‘*They are missed by existing services, not missing . . . young people whose needs are not met by the current, at times, arbitrary design of existing services*’ (ID55, Service provider). Future work should examine other populations and focus on service mapping to identify service-level characteristics that contribute to missing middle service gaps. Subsequent work may involve taking a social determinants health lens to engaging socially excluded groups in co-designing and reforming care systems and prevention initiatives (Simmons et al., 2021). Finally, participants suggested developing a range of labels to differentiate various groups with unmet care needs. For example, the suggestion was made to label those ineligible for Medicare services in Australia the ‘*invisibles*’ (ID46, Clinician). There was a clear desire to further examine other existing service gaps and to develop shared language and consensus descriptors for other unmet needs in mental health beyond the foci of this study.

### Limitations

The proposed definition was developed within an Australian context. Some elements may be generalisable, but the definition may lack utility in extrapolation to different countries and cultures. Furthermore, the study focused on developing a definition of the missing middle within the context of youth mental health and the conceptualisation may differ for population groups in other life stages. Despite this, the framing of the concept as a service gap, and shifting the focus away from the individuals who are affected by system failures, may be relevant in wider contexts even when the description of the nature of this service gap may need to be adapted for local contexts.

Attrition between Stages 2 and 3 of the Delphi is a consideration in interpreting the results; 37.5% of participants dropped out between these stages, which may have influenced the results during re-rating. The highest dropout rates were in the clinical (50%) and HS (39%) expert groups, whereas there was relatively high retention among participants with lived experience (15% drop-out rate). Participants were located across Australia; however, the lived experience, HS and clinical expert groups had substantially more Victorian participants (42%, 38% and 30%, respectively in Stage 1) posing a risk of bias. Future research should aim to recruit a representative sample for validation and to explore acceptability of the consensus definition more widely.

While the Delphi process facilitated refinement of the concept, results show expert groups agree the ‘missing middle’ is a multifaceted construct with multiple different concepts of relevance. This hampered the development of a simple, operational definition. We acknowledge the current definition is lengthy, which may affect its utility; however, trustworthiness and rigour in accurately reflecting results in qualitative research are paramount. This research is a first step towards developing a definition in the youth mental health context and further validation and refinement of the definition may be necessary.

Finally, semantic change, shifts in the core meaning of words, are often influenced by cultural or contextual changes (Haslam, 2016). As care needs, environmental stressors, policies and mental health systems change over time, the way in which the missing middle is conceptualised will continue to evolve. Given the importance of accurately capturing up-to-date definitions of the missing middle service gap (and service gaps at other levels, e.g. the ‘low intensity gap’; Productivity Commission, 2020), further research is recommended.

## Conclusion

This study shows agreement across expert groups to position the missing middle as a structural issue: ‘*This term does not usually describe the needs of the young person, it refers to what they are missing out on which is proper, effective treatment*’ (ID131, HS expert). Findings support replacing the term ‘missing middle’ with ‘missing middle service gaps’. The positioning clearly places responsibility onto service systems to ensure equitable access for youth who are ‘*not receiving the level of services that are transformational for their recovery*’ (ID134, Carer). Results also suggest that a heterogeneous group of individuals are affected by the missing middle service gap. Future research to map healthcare service systems is essential for strengthening the evidence regarding missing middle service gaps and illuminating how best to address these care gaps.

## Supplemental Material

sj-docx-1-anp-10.1177_00048674241299221 – Supplemental material for The missing middle service gap: Obtaining a consensus definition of the ‘Missing Middle’ in youth mental health Supplemental material, sj-docx-1-anp-10.1177_00048674241299221 for The missing middle service gap: Obtaining a consensus definition of the ‘Missing Middle’ in youth mental health by Jana M Menssink, Caroline X Gao, Isabel Zbukvic, Sophie Prober, Athina Kakkos, Alice Watson, Sue M Cotton and Kate M Filia in Australian & New Zealand Journal of Psychiatry
