# Supplementary material for: The missing middle service gap: Obtaining a consensus definition of the ‘Missing Middle’ in youth mental health
Source: Aust N Z J Psychiatry. 2024 Nov 27;59(2):152–61. doi: 10.1177/00048674241299221 (PMC11783981; doi:10.1177/00048674241299221)

# Supplementary Figure 1

Participants were provided with a Feedback Report consisting of a visual graph showing their own previous response in comparison to the other groups' responses (see excerpt below). They were provided with the opportunity to change their response or keep their rating the same as in the previous round.


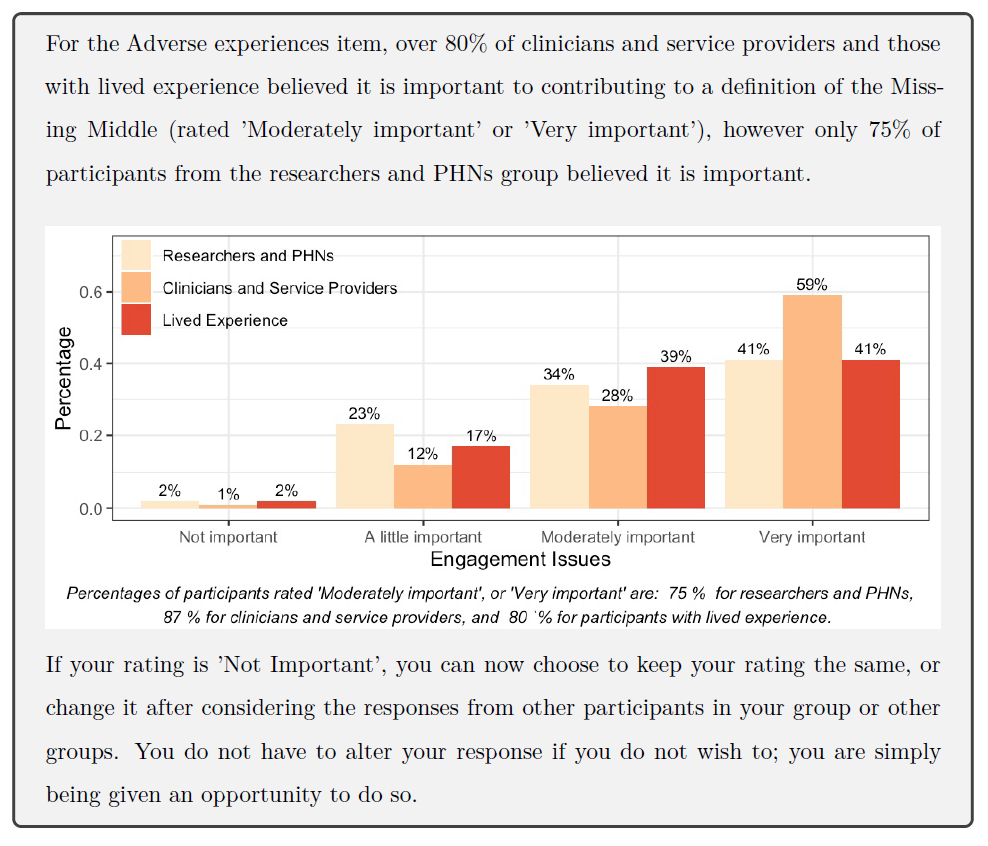

Supplement: sj-docx-1-anp-10.1177_00048674241299221 – Supplemental material for The missing middle service gap: Obtaining a consensus definition of the ‘Missing Middle’ in youth mental health [file sj-docx-1-anp-10.1177_00048674241299221.docx]
